# Protein-Protein Interactions: Gene Acronym Redundancies and Current Limitations Precluding Automated Data Integration

**DOI:** 10.3390/proteomes1010003

**Published:** 2013-05-31

**Authors:** Juan Casado-Vela, Rune Matthiesen, Susana Sellés, José Ramón Naranjo

**Affiliations:** 1 Spanish National Research Council (CSIC) - Spanish National Biotechnology Centre (CNB), Darwin 3, Cantoblanco, 28049 Madrid, Spain; E-Mail: naranjo@cnb.csic.es; 2 Institute of Molecular Pathology and Immunology (IPATIMUP), University of Porto, Rua Dr. Roberto Frias, 4200-465 Porto, Portugal; E-Mail: rmatthiesen@ipatimup.pt; 3 Alicante University, San Vicente del Raspeig Campus, 03690 Alicante, Spain; E-Mail: susana.selles@ua.es; 4 Centro Investigación Biomédica en Red (CIBERNED, Neurodegenerative disorders), Darwin 3 28049 Madrid, Spain

**Keywords:** bioinformatics, calsenilin, choline kinase, data integration, DREAM, gene acronym, gene redundancy, HGNC, HUGO, human interactome, KChIP3, protein accession, protein interactions, protein-protein prediction, uromodulin

## Abstract

Understanding protein interaction networks and their dynamic changes is a major challenge in modern biology. Currently, several experimental and *in silico* approaches allow the screening of protein interactors in a large-scale manner. Therefore, the bulk of information on protein interactions deposited in databases and peer-reviewed published literature is constantly growing. Multiple databases interfaced from user-friendly web tools recently emerged to facilitate the task of protein interaction data retrieval and data integration. Nevertheless, as we evidence in this report, despite the current efforts towards data integration, the quality of the information on protein interactions retrieved by *in silico* approaches is frequently incomplete and may even list false interactions. Here we point to some obstacles precluding confident data integration, with special emphasis on protein interactions, which include gene acronym redundancies and protein synonyms. Three human proteins (choline kinase, PPIase and uromodulin) and three different web-based data search engines focused on protein interaction data retrieval (PSICQUIC, DASMI and BIPS) were used to explain the potential occurrence of undesired errors that should be considered by researchers in the field. We demonstrate that, despite the recent initiatives towards data standardization, manual curation of protein interaction networks based on literature searches are still required to remove potential false positives. A three-step workflow consisting of: (i) data retrieval from multiple databases, (ii) peer-reviewed literature searches, and (iii) data curation and integration, is proposed as the best strategy to gather updated information on protein interactions. Finally, this strategy was applied to compile *bona fide* information on human DREAM protein interactome, which constitutes liable training datasets that can be used to improve computational predictions.

## 1. Introduction

In 1996, it was estimated that the human genome consists of 50,000–100,000 genes [1]. Far from the previous estimations, and in the light of the results from human sequencing projects [2,3], the current number of human protein-coding genes includes exactly 20,484 different entries [4] [updated from the HUGO Gene Nomenclature Committee (HGNC), May 2013]. The human genome is considered to be almost completely finished, meaning that the final number of protein-coding genes is not likely to increase significantly in the future and that the canonical sequence of each protein-coding gene is known. In parallel, the sequences of 20,249 different human protein products (the longest isoform produced after translation of each protein-coding gene) are also available (UniprotKB/ Swiss-Prot, May 2013). On the one hand, the wealth of information available offers unprecedented opportunities towards data integration at the two different levels (genomics and proteomics). On the other hand, the bulk of information available seems insufficient to explain the complexity of humans. In this sense, the identification of protein interactions and their regulation may help to unravel novel and relevant information. This hypothesis relies on the fact that proteins do not exist as isolated entities inside cells, but perform their function(s) through the interaction with other molecules. 

The size and dynamics of the human interactome is currently unknown. As displayed in Table 1, different estimations on the number of protein-protein interactions in humans widely differ, ranging from ~13,000 to ~370,000. Even the definition of the term “protein interaction” significantly varies among references (binary interactions or protein complexes, stable interactions *versus* weak or transient, predicted computational interactions without experimental validation may be considered, *etc.*). 

Although there is hardly a consensus on the number and validity of protein interactions, there is ample agreement on the idea that our understanding of the human interactome is in its early stages. Deciphering the interacting network of each individual protein and the conditions altering the interactome may be crucial to understanding their function and thus requires the integration of data available gathered from different sources including: (i) experimental data, (ii) prediction algorithms, (iii) database searches, and (iv) literature searches.

**Table 1:** Different estimations of the human interactome published in the literature including references and a brief description of the interactions.

Human interactome [reference] publication year	Description
375,000 [5] 2005	These authors used literature-mining algorithms and then estimated the number of protein interactions assuming 25,000 human genes.
154,000–369,000 [6] 2006	The authors quoted that their estimation includes protein complexes.
650,000 [7] 2008	This estimation relies on data retrieved from Y2H ^a^ experiments and database searches.
130,000 [8] 2009	This number of protein interactions exclusively considered binary interactions.
13,217 ^b^ [9] 2012	This estimation considered the longest protein isoform ^c^ of 20,846 human protein sequences. The size of the interactome was estimated using computational methods based on structural inference. The authors claimed that this estimation also includes self-interactions.

^a^ Y2H: yeast-two-hybrid; ^b^ Includes self-interactions and it is based on structural inferences; ^c^ See [10] for definitions on protein isoform and protein species.

Regarding experimental data, three main experimental strategies allow for large-scale screening of protein networks, including yeast-two-hybrid (Y2H) experiments, co-immunoprecipitation or affinity purification, followed by mass spectrometry and protein arrays [11,12]. The biochemical principles underlying each experimental approach are different. Thus, it is well acknowledged that the three strategies may lead to the identification of different subsets of protein partners. For example, immunoprecipitation strategies lead to the identification of both directly and indirectly associated proteins (e.g., protein complexes) [11], whereas protein arrays typically focus on direct (also termed binary or physical) protein interactions [12]. Accordingly, multiple experimental approaches may be performed to screen for protein interactions and the results should be considered as complementary.

A number of prediction algorithms and computational methods currently coexist and can be used to infer the occurrence of protein–protein interactions [13,14,15,16,17,18,19]. These algorithms rely on one or more features—such as genomic sequence, topological genomic clustering, protein sequence, protein structure, protein functional/structural domains or evolutionary relationship—and may also take advantage of known protein–protein interaction datasets to test, train, and improve the quality of their predictions. A comparative overview of prediction algorithms is beyond the scope of this report, but it is important to underline that computational algorithms frequently take advantage of reliable training datasets (*i.e*., *bona fide* list of protein interactions) to test and to improve their predictions. 

A range of databases currently allows the retrieval of information on protein interactions, including predictions of interactions and even modeling the pathways involved (reviewed in [20]). In this regard, it is important to distinguish databases compiling data from experimentally observed protein interactions from those reporting predictions without experimental evidence [21]. Another issue for consideration is that the list of candidate protein partners retrieved may significantly differ among databases, due to the fact that the amount and quality of the information deposited in each database are not really comparable ([22] reviewed protein-protein interaction databases and their sources of information). The overall information overlap among databases is limited and, thus, gathering information from as many databases as possible may represent an advantage if thorough information on the interactome of a specific protein is the objective. This task currently constitutes an obstacle that may be prohibitive in terms of time. In order to facilitate interaction data retrieval from multiple databases, several web-based search engines such as PSICQUIC [23], DASMI [24] and BIPS [25] were recently developed and made publicly available for the scientific community. These web tools significantly simplify the screening of information. Nevertheless, relevant information affecting protein interactions is frequently overlooked [26] and, more importantly, still suffers from high rates of false positives and errors [9]. For that reason, literature searches on peer-reviewed journals still constitutes the main source of information on protein-protein interactions in two ways: first, literature searches may retrieve information that may not be included in databases and, second, the comparison of the list of protein interactors retrieved upon database searches and after literature searches enables manual correction of potential errors. To exemplify this, a recent report focusing on the identification of the *Salmonella* host interactome [26] compared the information available in >100 databases and >2,200 journal articles, leading to the identification of 62 protein-protein interactions of *Salmonella* proteins with human and mouse proteins, both acting as host for this bacteria. The authors stated that only six of these interactions were retrievable from databases and concluded that literature searches represented an essential step. In this sense, we strongly support that manual curation of protein-protein interactions based on literature searches is a requisite [27]. Indeed, in this report we point to several constraints hindering the fast integration of information on protein interactions, which support the need for curation strategies based on literature data. We also point out the main sources of errors that preclude fully automated protein-protein data retrieval: the proper use of standard acronyms, and the occurrence of acronym redundancies and synonyms that are arbitrarily used in the literature. 

## 2. Experimental

### 2.1. Human Gene Consensus Sequences and Acronyms

The updated list of human genes was adapted from the information provided by the National Center for Biotechnology Information [28]. This information is provided in this report as a comma separated values (CSV) (Supplementary File 1) and is also available in our public data repository at [29]. This file includes the complete list of protein-coding genes (20,484 entries) and pseudo genes (13,191 entries). 

### 2.2. Human Protein Consensus Sequences and Acronyms

The consensus amino acid sequence of human proteins was retrieved from the Uniprot protein knowledgebase/Swiss-Prot [30]. The strategy used to retrieve this information consisted of the inclusion of the following string of keywords and Boolean connectors in the “query” field of this database: organism: 9606 AND keyword: “Complete proteome [KW-0181]” reviewed:yes. This string of characters retrieved 20,249 different human protein entries (May 2013) and can be used *ad hoc* for the retrieval of the updated list of human proteins. The list of proteins considered in this report is provided as a comma separated values (CSV) (Supplementary File 2) and can be downloaded from our public data repository at [29]. Relevant information on the proteins such as protein description, Swiss-Prot protein accessions and entries are also included. Protein accessions and entries represent unique and unambiguous identifiers for each protein (see [31] for further information).

### 2.3. Database Searches and Protein Networking

Protein networks and list of interacting proteins were carried out using PSICQUIC [23], DASMI [24] and BIPS [25]. These three search engines are representative examples of popular web-based tools frequently used by researchers in the field and able to merge protein interaction data combining the information deposited in multiple databases. 

### 2.4. Gene Redundancy

The official gene acronyms and the corresponding synonyms accepted for each human gene were retrieved from UniProtKB (May 2013). Redundant symbols within gene names and synonyms were retrieved for each UniProtKB entry using case-sensitive comparison of all human symbols. A text file where each line represents alternative symbols used for a specific gene was prepared. For each line, redundant gene symbols were removed. That is, each line contains a non-redundant list of alternative symbols for a specific gene. From this per gene entry non-redundant list, a new redundant list of symbols was prepared where each line contains one symbol. The frequency of each symbol in this redundant list corresponds to the number of times a symbol refers to different genes and can conveniently be calculated using the “table” command in the R statistical programming language.

## 3. Results and Discussion

### 3.1. Evidence for Erroneous Protein-Protein Interactions after Database Searches

**Figure 1:**
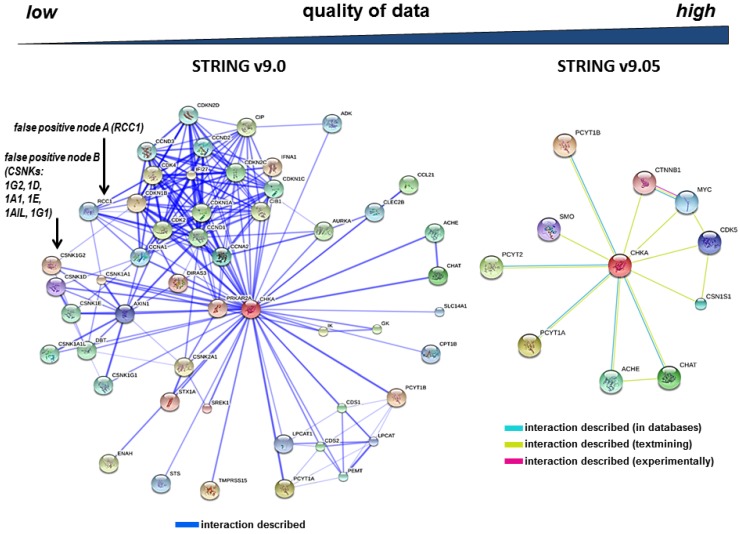
Protein interaction network corresponding to human choline kinase (CHKA) using two different versions of STRING [34] versions 9.0 and 9.05. String may be accessed through it web interface [35] or selecting the corresponding option in PSICQUIC View [36]. Searches were triggered using the Swiss-Prot accession number P35790 [32], which uniquely identifies CHKA. The query protein (CHKA, depicted as a red sphere) appears connected with surrounding candidate interacting proteins. Left panel: database searches using STRING v9.0 retrieved false positive nodes A (RCC1, regulator of chromosome condensation) and B (casein kinase proteins -CSNKs- 1G2, 1D, 1A1, 1E, 1AIL and 1G1). Right panel: a recent version of the software (STRING v.9.05) removed false positives and improved the quality of CHKA interactions. STRING also shows information on the source of the interaction mapped is also included as colored lines (databases, textmining and experimental evidence). The default scoring filtering criteria were selected in all cases.

In this report, we demonstrate that searching for protein–protein interactions using *in silico* searches may lead to errors. To exemplify this, we first retrieved for human choline kinase (gene acronym: CHKA, accession: P35790, entry: CHKA_HUMAN) interactors using three web interfaces able to retrieve and integrate data from multiple source databases: PSICQUIC [23], DASMI [24] and BIPS [25]. In all cases, database searches were triggered using the human choline kinase Swiss-Prot accession (P35790, [32]).The Swiss-Prot accession acts as a unique and unambiguous identifier corresponding to a single protein product. Surprisingly, despite the unambiguity of the accession number submitted to the searches, we noticed that the three web engines listed one or more false protein interactors. As depicted in Figure 1 and Supplementary File 3, the list of wrong CHKA interactors included proteins such as regulator of chromosome condensation (gene acronym: RCC1, accession: P18754, entry RCC1_HUMAN) [33] and several casein kinases (CSNKs). Such errors could only be confirmed upon manual curation using experimental data published in peer-reviewed journals. Fortunately, information and/or links to citations containing original data are typically provided through search engines (see Supplementary File 3), facilitating their access to researchers.

The example above clearly evidences that database queries may lead to errors, thereby allowing us to hypothesize that the source for such errors is the lack of consensus in the gene acronyms and protein abbreviations used to refer to human proteins. This hypothesis is based on the observation that casein kinases and choline kinase may be abbreviated in the scientific literature using the same abbreviation (*i.e*., CK). This was already detailed in a recent publication [37], quoting that up to five different abbreviations may be used to refer to the human choline kinase alpha gene (CHKA, CHK, CKI, CK and EK) and they are all accepted as synonyms and are simultaneously and arbitrarily used in the literature. The lack of consensus in the use of gene acronyms may add confusion when searching for protein interactions. CHKA is exclusively attributed to choline kinase genes, CHK and CKI can be used to denote checkpoint kinases or casein kinase I, respectively. CHK may also refer to megakaryocyte-associated tyrosine kinase (CHK) and the three letter code “CHK” is also included in the gene symbol used for choline kinase beta (CHKL). Similarly, two letter symbols (CK and EK) are included in a number of genes within the list, which contributes to add some noise. 

From the example above, it becomes obvious that the same string of characters used as abbreviations or acronyms may refer to different genes or gene products. We also demonstrate that such redundancy may lead to the inclusion of erroneous candidates among the list of protein interactors.

Database builders and data integration initiatives are very aware that gene acronym redundancy may significantly undermine the quality of the list of interactions retrieved by *in silico* approaches. Indeed, as depicted in Figure 1, different versions of the same software may significantly differ since both data annotation and curation improve with time. In the absence of consensus, and given that multiple synonyms currently coexist in the literature, data curation is required. In this sense, ways to report the occurrence of errors from users to database builders should be implemented because the contribution from users may significantly foster the tedious task of data curation.

### 3.2. The Redundancy of Gene Acronyms

**Figure 2:**
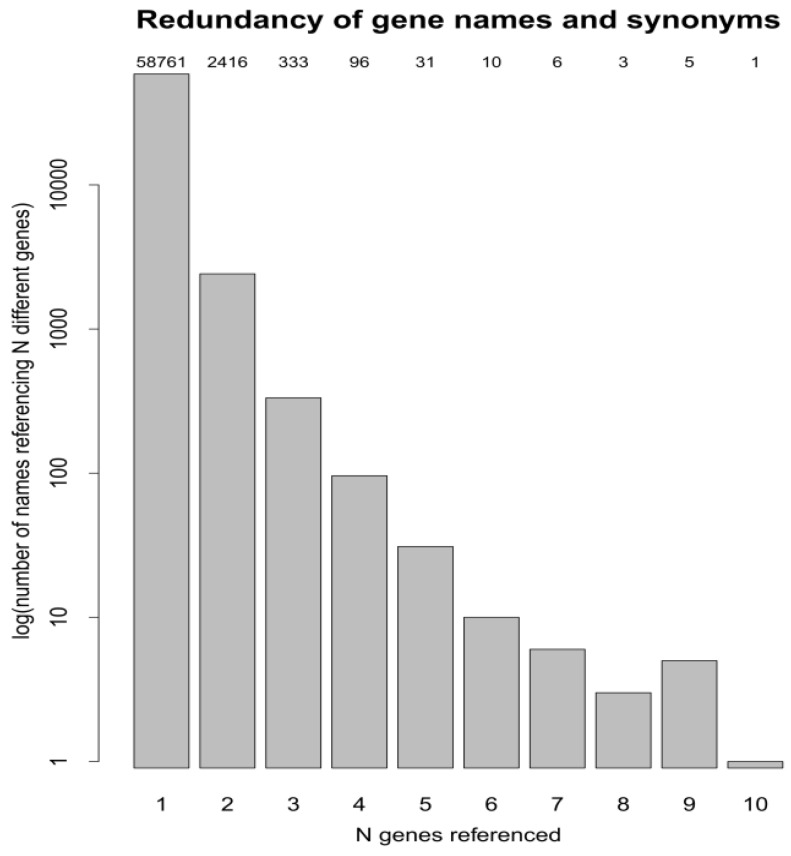
Bar-plot demonstrating the redundancy displayed by human gene acronyms and their synonyms.

As quoted in the example above, one gene acronym may refer to different genes or gene products and such redundancy may lead to errors when identifying protein-protein interactions through automated database searches. Thus, we hypothesized that redundancy leads to ambiguity that constitutes the causal origin of mistakes, introducing erroneous protein interactors through *in silico* searches. This fact raised the question whether and to what extent such redundancy could be extrapolated to other proteins of interest. Therefore, we were prompted to investigate the frequency of gene acronym redundancy and its effect on the identification of protein–protein interactions. To that aim, we built a file (Supplementary File 1) containing the description and the gene acronyms of the 20,484 human protein-coding genes. It is important to note that acronyms from the nomenclature authority and synonyms of each gene found in the literature are also included in the same file. All the acronyms and synonyms listed in Supplementary File 1 are accepted and arbitrarily used by authors in the literature. Therefore, this file likely constitutes the best resource for measuring the frequency of redundancy. We calculated the redundancy of gene names and synonyms and plotted the number of names referencing N different genes *versus* N genes referred (Figure 2).

Interestingly, as displayed in Figure 2, the repetition of gene acronyms is a frequent event and a significant portion of the genes displayed redundancies ranging from two, up to ten. This plot evidences that one gene acronym may designate multiple genes and/or protein. Importantly, a significant percentage of gene acronyms may refer to two or more different genes. The gene acronym PPIASE constitutes a paradigmatic example of gene acronym redundancy since this is used to abbreviate the name of ten different protein-coding human genes (GeneIDs: 2080, 2081, 2086, 2287, 2288, 2289, 8468, 11328, 51645, 51661 and 60681, all of them mapped on different gene locations) (Supplementary File 4). Obviously, such redundancy leads to undesired ambiguities and errors introduced by database retrieval algorithms, which are unable to discern the attribution of a gene acronym to a certain gene or protein product. As explained above, the lack of consensus regarding the use of gene symbols may constitute a significant hurdle in the process of retrieving *bona fide* protein interactions from public repositories. On the other hand, using gene symbols to trigger searches in protein interaction repositories is a common practice. Moreover, the string of characters “PPIAase” is also found in the description of a range of different human genes (see Supplementary File 4), contributing to extra confusion.

As a result, we show that the probability of including false protein interactions after *in silico* searches using gene symbols is relatively high. This means that it is probable that using gene abbreviations as the only information included in database searches may end up showing interactions that do not strictly correspond to the protein of interest. Consequently, in order to discard potential errors, manual curation of the list of interactors retrieved using gene symbols against published data seems highly recommendable, especially in those cases where the frequency of the gene symbols used for a protein in the literature is ≥2.

Regarding proteins, in a previous section using choline kinase as an example, we showed that one protein may correspond to multiple gene acronyms. As a means to preclude such ambiguity, the UniprotKB/Swiss-Prot database uses two different types of alphanumerical identifiers for each protein entry: accession and entry names [38]. Accessions are composed of six consecutive alpha-numerical characters without spaces or special characters and constitute stable and unique identifiers for each protein. These are stable identifiers and should be used to cite UniprotKB/Swiss-Prot entries. Upon integration into UniprotKB/Swiss-Prot, each entry is assigned a unique accession, which is called “Primary (citable) accession.”

Probably the best way to circumvent the ambiguity problem caused by gene acronym redundancies would be using consensus lists of gene acronyms exclusively attributed to single genes (not shared by any other gene) [4]. Nevertheless, as exemplified above, manual curation of metadata seems necessary to preclude unnecessary errors. In the meantime, we provide here an open-access standalone software tool termed “Gene Symbol Redundancy Checker” to facilitate validation of gene redundancies [39]. This software runs under Windows (32-bit or 64-bit systems, downloadable as zip compressed file) and calculates the degree of symbol redundancy for a list of gene symbols pasted into the application. This can, for example, be a list of genes in a network provided by an online tool. The output also summarizes all alternative symbols for all the genes that share a given gene symbol. The output can be subsequently exported or copy and pasted to any spreadsheet data-processing software for further analysis.

### 3.3. Protein Accessions, Protein Entries and Database Searches

Several databases may be used for proteomic research and their selection mainly depends on the quality of protein annotation and on objective pursued. In this regard, the Swiss-Prot protein database is widely considered as the “gold standard” for proteomic approaches because it contains high-quality and manually annotated data, as well as the sequence of the proteins and access to protein-protein interaction data. Remarkable efforts were carried out in the past and others are under way towards the convergence, integration and standardization of the bulk of the data available in different protein databases. For instance, in 2011, the Swiss-Prot database incorporated the information of the human International Protein Index database (further information in this link [40]), a database frequently used in the past decade for proteomic experiments. It is evident, therefore, that protein databases rapidly evolve. Improved versions are continuously released and the descriptions of the proteins included in them also vary with time. In the case of the Swiss-Prot database, such changes are listed in The UniprotKB/Swiss-Prot Sequence/Annotation Version Archive (UniSave).

For that reason, every protein included in the Swiss-Prot database is characterized by at least one protein accession and one protein entry that are exclusively attributed to a known protein product. Proteins characterized by more than one accession may be included in Swiss-Prot. For example, uromodulin (gene acronym: UMOD, accession: P07911, entry: UROM_HUMAN) was associated in the past with four different Swiss-Prot accessions (P07911, Q540J6, Q6ZS84 and Q8IYG0, Swiss-Prot v57.15). Currently, only the first accession is accepted (P07911), but previous accession numbers still appear in the literature, contributing to the confusion. Regarding protein descriptions and continuing with the same example, uromodulin is also frequently referred to as the Tamm-Horsfall protein. The latter term is frequently used in medical literature for historical reasons because it was first purified from the urine of healthy individuals by Tamm and Horsfall [41,42]. It is important to underline that the two descriptions are correct and synonymous, but their arbitrary use in the literature complicates the integration of data and may hamper thorough retrieval of protein interactions. 

The trend towards data simplification and integration is evident, and the number of initiatives aiming overall integration of -omic(s) data is under way, but it is still not sufficient. As an example, a recent web-based application termed KUPKB (Kidney and Urinary Pathway Knowledge Base) offers access to data from multiple -omic approaches [43]. To reiterate, the lack of consensus to name genes and proteins significantly obstructs data integration. As a proof of principle, in a previous report, we built a consensus list of proteins found in human urine. This objective required the integration of proteomic metadata previously published and the combination of a list of proteins identified independently in different laboratories during the last decade [44]. Previously, we demonstrated that integrating and updating previous metadata may be a tedious task but it is necessary for data integration.

Accordingly, in order to promote automated data integration, the lists of proteins identified by proteomic experimental approaches should include primary accessions and/or entries exactly in the form they appear in the Swiss-Prot database and, if possible, the corresponding gene acronym as recommended by the HUGO Gene Nomenclature Committee. Similarly, the use of recommended protein names exactly as they appear in the Swiss-Prot database should be promoted, since this could significantly simplify database searches whilst reducing ambiguity at the same time.

### 3.4. Database Searches Fail to Include Dynamic Changes on Protein Networks

The retrieval of the overall list of interactors for a specific protein of interest is essential to discerning novel from previous findings and also serves to identify the pathways involved. In the sections above we quoted some problems affecting the number and quality of interactors retrieved by searching in multiple databases and the need to complete and curate that information by comparing the list of interactors with published literature. However, the interactome of a given protein of interest is not static and adapts to changes in the environmental conditions. Thus, understanding the dynamics or protein interaction networks is crucial to unraveling the role and the regulation of proteins under different cellular conditions [45]. Here, we briefly point to the fact that *in silico* database searches typically fail to provide information on protein interaction changes as a response to modifications in the experimental conditions. To exemplify this, we used human calsenilin (gene acronym: KCNIP3, accession: Q9Y2W7, entry: CSEN_HUMAN), a protein of interest in our lab that was originally identified as calsenilin—a Ca^2+^-binding protein belonging to the family of neuronal calcium sensor proteins [46]. Shortly thereafter, this protein was found to be identical to the Ca^2+^-dependent gene silencer DREAM (downstream regulatory element antagonist modulator) [47] and, later, to one of the interacting proteins (KChIPs)—or β-subunits—of the voltage-gated Kv channels, KChIP3 [48]. The three descriptions correspond to the same protein, since it is the product of a single gene, and localizes to three different cellular compartments (membrane, cytoplasm and nucleus). In these three cellular locations, DREAM plays different roles (K^+^ channel, Ca^+2^ binding protein and transcriptional repressor, respectively) and interacts with different subsets of molecules—including the interaction with DNA in the nucleus [49]. At structural level, DREAM harbors four EF-hand domains able to bind Ca^+2^ ions that provoke structural changes. Thus, it is foreseeable that the DREAM interactome significantly varies depending on sub-cellular localization or the presence/absence of Ca^+2^. It is important to underline that none of the databases tested—including 22 databases focused on protein-protein interactions available at [22,50] or integrated web-based platforms like PSICQUIC [23], DASMI [24] and BIPS [25]—offered direct information about DREAM interactome changes or provided clues on factors modifying or affecting DREAM networking. Conversely, a good deal of information can be extracted from published literature (for review see [51]). Thus, we decided to compile the current status of DREAM interactome (Table 2) including supporting references, year of publication, *in vitro* and *in vivo* models used in the experiments, human gene acronyms (including synonyms), UniprotKB/Swiss-Prot accessions and entries, description, techniques used for detection of the interactions. Further information influencing DREAM interactions is also included, such as the potential effect of Ca^+2^ and post-translational modifications.

**Table 2:** DREAM interactome: publication year, experimental model used, corresponding human gene acronyms (May 2013), Swiss-Prot protein accessions release 2013_05 (May-2013), protein entries, protein descriptions and synonyms, detection method used and other additional relevant information is also included.

Year Ref.	Experimental model used	HUMAN GENE ACRONYM HUGO acronym (bold), other synonyms	Human Swiss-Prot accession, entries	Protein description and synonyms	Detection method	Additional information
1998 [46]	*in vitro*: Y2H, PSEN as bait and human brain cDNA. *in vitro*: COS-7 cells.	**PSEN1**,FAD, PS1, S182, PS-1, AD3	P49768 PSN1_HUMAN	Presenilin-1, Protein S182.	Y2H and co-precipitation followed by Western blot.	Y2H experiments included in this report could not confirm PSEN2–DREAM interaction.
1998 [46] 2008 [52]	*in vitro*: Y2H, PSEN as bait and human brain cDNA. *in vitro*: COS-7 cells. *in vitro*: SH-SY5Y cells.	**PSEN2**, AD3L, PS2, STM2, AD4, CMDV1	P49810 PSN2_HUMAN	Presenilin-2, AD3LP, AD5, E5-1, STM-2.	Co-localization in COS-7 cells and co-precipitation followed by Western blot. Co-transfection, co-precipitation followed by Western blot	Y2H experiments were not able to confirm PSEN2-DREAM interaction. This interaction occurs in a Ca^+2^-independent manner.
1996 [47] 1999 [53] 2011 [54]	*in vitro*: CHO cells. *in vitro*: HEK 293 cells. *in vitro*: CHO cells.	**KCNIP3**, CSEN, DREAM, KCHIP3	Q9Y2W7, CSEN_HUMAN	Calsenilin, A-type potassium channel modulatory protein 3, DRE-antagonist modulator (DREAM), Kv channel-interacting protein 3 (KCHIP3)	Molecular mass from SDS-PAGE gels and Western blot.	Multimeric forms (monomers [47], dimers [47,54] and tetramers [47,53] described).
2000 [53]	*in vitro*: HEK293, NB69, SK-NMC cells.	**CREM**, CREM-2, ICER, hCREM-2	Q03060 CREM_HUMAN	cAMP-responsive element modulator, inducible cAMP early repressor (ICER).	Pull-down using CREM as bait.	DREAM–CREAM protein-protein interaction leads to loss of binding of the transcriptional repressor DREAM to target genes [53].
2000 [48]	*in vitro*: Y2H, KCNIP4 as baits.	**KCNIP4**, CALP, KCHIP4	Q6PIL6 KCIP4_HUMAN	Kv channel-interacting protein 4 (KChIP4), A-type potassium channel modulatory protein 4, Calsenilin-like protein, Potassium channel-interacting protein 4.	Y2H and co-precipitation.	Y2H cDNA library was constructed from polyA^+^ RNA extracted from rat brain.
2002 [55]	*in vivo*: rat brain nuclear extract. *in vitro*: HEK293, PC12 cells.	**CREB1**, CREB	P16220 CREB1_HUMAN	Cyclic AMP-responsive element binding protein 1.	Immunoprecipitation from brain nuclear extracts using anti-DREAM antibody.	DREAM–CREB1 protein-protein interaction prevents recruitment of CBP by phospho-CREB and affects CRE-dependent transcription.
2004 [56]	*in vivo*: thyroid-derived FRTL-5 cells. *in vitro*: co-immunoprecipitation in CHO cells.	**NKX2-1**, BCH, BHC, NK-2, NKX2.1, NKX2A, TEBP,TITF1,TTF-1, TTF1	P43699 NKX21_HUMAN	Homeobox protein Nkx-2.1, Homeobox protein NK-2 homolog A, Thyroid nuclear factor 1, Thyroid transcription factor 1.	Co-precipitation using GST-DREAM as bait in FRTL-5 thyroid-derived cells.	DREAM regulates the expression of the thyroglobulin gene.
2005 [57] 2008 [58]	*in vitro:* co-incubation query protein (GST-DREAM) and bait protein (6His-VDR).	**VDR**, NR1/1	P11473 VDR_HUMAN	Vitamin D3 receptor, 1,25-dihydroxyvitamin D3 receptor, Nuclear receptor subfamily 1 group I member 1.	Pull-down after incubation of Ni-Sepharose beads with a 1:1 protein mixture of GST-DREAM (Δ^65-256^) and 6His-VDR.	Ca^2+^ induces dimerization of DREAM and a binding interaction between DREAM and VDR. Chromatin immunoprecipitation showed that DREAM also binds to DNA, acting as a transcriptional regulator on vitamin D and retinoic acid response elements.
2006 [59]	*in vitro*: Y2H, DREAM as bait. *in vitro*: co-immunoprecipitation in H4 cells.	**CtBP1**,CTBP	Q13363 CTBP1_HUMAN	C-terminal-binding protein 1.	Y2H using N-terminus of DREAM as bait and co-precipitation.	DREAM–CTBP may modulate transcriptional repression of c-fos.
2006 [59]	*in vitro*: Y2H, DREAM as bait. *in vitro*: co-immunoprecipitation in H4 cells.	**CtBP2**, ribeye	P56545 CTBP2_HUMAN	C-terminal-binding protein 2.	Y2H using N-terminus of DREAM as bait and co-precipitation.	DREAM–CTBP may modulate transcriptional repression of c-fos.
2007 [60]	*in vitro*: Y2H, DREAM as bait. *in vitro*: co-immunoprecipitation in PC12 cells. *in vitro*: DREAM Ser95 phosphorylation using HEK293.	**GRK6**, GPRK6	P43250 GRK6_HUMAN	G protein-coupled receptor kinase 6, G protein-coupled receptor kinase GRK6.	Y2H and confirmed by co-precipitation of PC12 cell extracts and antibodies specific for GRK6.	A mutated DREAM insensitive to Ca^+2^ was used to preclude potential artifacts in Y2H screening.
2007 [60]	*in vitro*: Y2H, DREAM as bait. *in vitro*: co-immunoprecipitation using PC 12 cells. *in-vitro*: DREAM Ser95 phosphorylation using HEK293.	**ADRBK1**, BARK1, BETA-AEK1,GRK2	P25098 ARBK1_HUMAN	Beta-adrenergic receptor kinase 1, G-protein coupled receptor kinase 2.	Co-precipitation of PC12 cell extracts and antibodies specific for GRK6.	May regulate DREAM function through phosphorylation.
2008 [61]	*in vitro*: Y2H, mouse G3GALT2 as bait and human brain cDNA. *in vitro*: co-immunoprecipitation in CHO-K1 cells.	**B3GALT2**, BETA3GALT2, GLCT2, beta3Gal-T2	O43825 B3GT2_HUMAN	Beta-1,3-galactosyltransferase 2, Beta-1,3-GalTase 2, UDP-galactose:2-acetamido-2-deoxy-D-glucose 3beta-galactosyltransferase 2.	Y2H using N-terminus of GalT2 as bait.	DREAM is involved in the trafficking of glycosyl-transferases to Golgi and endoplasmic reticulum.
2009 [62]	*in vivo*: thyroid glands from mice. *in vitro*: co-immunoprecipitation in CHO cells.	**TSHR**, CHNG1, LGR3, hTHSR-1	P16473 TSHR_HUMAN	Thyrotropin receptor, Thyroid-stimulating hormone receptor.	Co-immunoprecipitation using mice thyroid protein extracts and cells transfected with hemagglutinin-tagged DREAM.	Activation of cAMP signaling pathway, thyroid enlargement and nodular development.
2010 [63]	*in vivo*: co-immunoprecipitation using mouse hippocampal extracts.	**DLG4**, PSD-95, PSD95, SAP-90, SAP90	P78352 DLG4_HUMAN	Disks large homolog 4, Postsynaptic density protein 95, Synapse-associated protein 90.	Co-immunoprecipitation.	DREAM modulates the function of postsynaptic NMDA receptor, synaptic plasticity, behavioral learning and memory.
2010 [64]	*in vivo*: co-immunoprecipitation using rat brain extracts.	**CACNA1H**, Cav3.2	O95180 CAC1H_HUMAN	Voltage-dependent T-type calcium channel subunit alpha-1H, Low-voltage-activated calcium channel alpha1 3.2 subunit, Voltage-gated calcium channel subunit alpha Cav3.2	Co-immunoprecipitation.	Rat brain protein extracts.
2010 [64]	*in vivo*: co-immunoprecipitation using rat brain extracts.	**CACNA1I**, Cav 3.3, KIAA1120	Q9P0X4 CAC1I_HUMAN	Voltage-dependent T-type calcium channel subunit alpha-1I, Voltage-gated calcium channel subunit alpha Cav3.3.	Co-immunoprecipitation.	Rat brain protein extracts.
2010 [65]	*in vitro*: co-immunoprecipitation in HEK293 cells. *in vivo*: co-immunoprecipitation in rat hippocampus extracts.	**GRIN1**, GluN1, NR1, NMDAR1, NMDR1 *	Q05586 NMDZ1_HUMAN	Glutamate receptor ionotropic, Glutamate [NMDA] receptor subunit zeta-1, N-methyl-D-aspartate receptor subunit NR1.	Immunoprecipitation from rat hippocampus extracts.	This interaction supports the role of DREAM in learning and memory.
2011 [66]	*in vitro*: Y2H, DREAM as bait and human brain cDNA. *in vitro*: co-immunoprecipitation in PC12 and HEK293 cells.	**UBE2I**, C358BE.1, P18, UBC9	P63279 UBC9_HUMAN	SUMO-conjugating enzyme UBC9, SUMO-protein ligase, Ubiquitin carrier protein 9 Ubiquitin carrier protein I, Ubiquitin-conjugating enzyme E2, Ubiquitin-protein ligase I, p18.	Y2H and co-immunoprecipitation of PC12 cell protein extracts.	Sumoylation regulates nuclear localization of DREAM. A mutated DREAM insensitive to Ca^+2^ was used to preclude potential artifacts in Y2H screening.
2011 [54]	*in vitro*: Y2H, Ca^+2^ insensitive DREAM as bait and human bone marrow cDNA. *in vitro*: co-immunoprecipitation in COS-7 cells.	**Prdx3**,AOP-1, AOP1, HBC189, MER5, PRO1748, SP-22, Prx-III	P30048 PRDX3_HUMAN	Thioredoxin-dependent peroxidereductase, mitochondrial, Antioxidant protein 1, HBC189, Peroxiredoxin III, Peroxiredoxin-3, Protein MER5 homolog.	Y2H and co-immunoprecipitation of CHO cell protein extracts.	Prdx3 is a mitochondrial protein. Unlikely to mediate the regulation of DREAM under basal conditions. The subcellular compartment where the redox regulation of DREAM *in vivo* takes place not yet characterized.
2012 [67]	*in vivo*: co-immunoprecipitation using rat brain extracts.	**CALM1**,CAMI, CPVT4,DD132, PHKD, caM,CALML2	P62158 CALM_HUMAN	Calmodulin.	Affinity capture followed by mass spectrometric identification of interacting proteins.	In the presence of Ca^+2^, DREAM binds to calmodulin. A list of proteins potentially binding to DREAM under Ca^+2^-dependent and independent conditions is included in [67].
2012 [67]	*in vivo*: co-immunoprecipitation using rat brain extracts.	**PPP3R1**, CALNB1, CNB, CNB1	P63098 CANB1_HUMAN	Calcineurin subunit B type 1, Protein phosphatase 2B regulatory subunit 1, Protein phosphatase 3 regulatory subunit B alpha isoform 1.	Affinity capture and mass spectrometric identification of interacting proteins.	In the absence of Ca^+2^, DREAM binds to calcineurin subunit-B. A list of proteins potentially binding to DREAM under Ca^+2^-dependent and independent conditions is included in [67 ].

## 4. Conclusions

The study of protein interactomes and the pathways involved is a major objective currently pursued. The overall understanding of protein networks requires gathering and merging information from multiple sources (large-scale experiments, multiple data repositories and published literature) but compilation of protein interactions still constitutes a major current bottleneck. Different methodologies currently enable the generation of large bulks of data, and a plethora of databases are continuously fed with novel data. It is important to highlight that despite the rapid advances towards data integration and standardization, integration of interaction data from *in silico* sources is an error-prone task and, thus, requires manual data curation. 

Here, we pointed to two sources of ambiguities that promote the accumulation of errors: gene acronym redundancies and protein description synonyms. We take advantage of several proteins of interest in our group to exemplify the occurrence of such ambiguities and to evidence that arbitrary ways to name human genes and proteins undermine fully automated and reliable data integration.

We emphasize that gene acronym redundancies constitutes a frequent event. A significant number of gene acronyms suffer some degree of redundancy, which probably constitutes the most significant hurdle towards data integration. In parallel, at the protein level, it is obvious that different authors refer to the same proteins using different acronyms arbitrarily chosen. These two sources of ambiguities lead to errors, especially when using bioinformatics and data-mining algorithms for the retrieval of protein interactions. 

Initiatives such as the Human Proteome Organization Proteomics Standards Initiative (HUPO PSI-MI) focused on the need for data format standardization and proposed the incorporation of controlled vocabularies to facilitate data exchange and integration (see [27] for review). Despite the ongoing efforts, the list of potential interactors retrieved through bioinformatic tools still lacks reliability and requires confirmation of data through literature searches. The last statement is especially true assuming that protein-protein interactions available in databases will be always behind state-of-the-art information described in the literature. Moreover, looking for interactions in the literature may be advantageous in several ways: first, it may provide valuable interactions not included in databases; second, it is the best way to find detailed information on the experimental conditions favoring the interactions and the techniques used to identify them; and, finally, scientific articles serve as a basis for data curation. 

We applied the recommendations provided in this report to explore the current status of the human DREAM interactome. Updated information on this protein is tabulated in this report, including the list of interactors retrieved from databases and confirmed upon manual data screening based on supporting peer-reviewed literature. The DREAM interactome can, thus, be further exploited for drug discovery approaches and serves as a *bona fide* “training-set” for future improvements of protein-protein prediction algorithms.

To summarize, we propose the use of standard unambiguous gene acronyms and/or protein accessions in published literature and in electronic data repositories as the best way to promote data integration and to minimize the possibility of errors in the task of retrieving protein-protein interactions from databases. The use of unique gene and protein identifiers should be promoted by editorials and, if possible, in a retrospective manner (by including suitable gene and/or protein descriptions and abbreviations in those articles previously published). In our opinion, this could be the best way to foster data integration while ameliorating the tedious task of manually reviewing, extracting data and filtering out errors based on published data. This rationale is supported by the fact that once published, it is hardly possible to include changes, including designation and abbreviation of genes and proteins. In our opinion, our proposal is certainly feasible, since a list of unambiguous gene acronyms and protein accessions are already available from the HUGO Gene Nomenclature Committee (HGNC) and UniprotKB/Swiss-Prot). A clear and concise specification of the source of gene description and abbreviation may facilitate future data integration strategies, including the identification of protein interactions. Therefore, we propose that the source of genes and/or proteins used and their corresponding abbreviations should be clearly quoted in publications, including sentences such as: “Human gene names, description and abbreviations used in this report follow the recommendations from the HUGO Gene Nomenclature Committee (HGNC),” or “Human protein names, description and abbreviations follow the recommendations from the UniprotKB/Swiss-Prot database.” 

## Electronic Supplementary Material

**Supplementary File 1.** Full list of human genes, acronyms and description included in the National Center for Biotechnology Information. The list includes protein-coding genes, pseudo genes and non-coding RNA. The updated list of genes can be downloaded using the file transfer protocol (FTP) from the NCBI at [68].

**Supplementary File 2.** Full list of human proteins, accession, entries and descriptions included in the Uniprot protein knowledgebase/Swiss-Prot (UniprotKB/Swiss-Prot). Each human protein contained in this database is represented by one unique UniProtKB/Swiss-Prot accession and one entry (for further information see [31]. The updated list of human proteins can be retrieved using the following strings in the ‘query’ field: organism:9606 AND keyword: “Complete proteome [KW-0181]” reviewed:yes.

**Supplementary File 3.** Human choline kinase protein-protein interactions retrieved in databases using three different web interfaces: PSICQUIC View [36], DASMI [69] and BIPS [70]. In all cases, the search was triggered by introducing the UniprotKB/Swiss-Prot accession P35790 [32] as unique identifier for human choline kinase. RCC1 (regulator of chromosome condensation) was included among the list of candidates in all cases (highlighted in red), but this protein is a false positive and constitutes an error of database searches, as derived from [33] reporting the interaction between RCC1 and casein kinase I. The list of candidate interacting proteins, accessions (where available), supporting publications and the source (database) of information are included.

**Supplementary File 4.** Paradigmatic example of gene acronym redundancy. The use of the gene acronym PPIASE (or PPIase) can be found in the literature to abbreviate the name often different protein-coding human genes (GeneIDs: 2080, 2081, 2086, 2287, 2288, 2289, 8468, 11328, 51661 and 60681) mapped on different gene locations. The string of characters 'PPIASE' is also included in the description of a range of different proteins (highlighted in red).

## References

[1] Schuler G.D., Boguski M.S., Stewart E.A., Stein L.D., Gyapay G., Rice K., White R.E., Rodriguez-Tome P., Aggarwal A., Bajorek E. (1996). A gene map of the human genome. Science.

[2] Lander E.S., Linton L.M., Birren B., Nusbaum C., Zody M.C., Baldwin J., Devon K., Dewar K., Doyle M., FitzHugh W. (2001). Initial sequencing and analysis of the human genome. Nature.

[3] Venter J.C., Adams M.D., Myers E.W., Li P.W., Mural R.J., Sutton G.G., Smith H.O., Yandell M., Evans C.A., Holt R.A. (2001). The sequence of the human genome. Science.

[4] Gray K.A., Daugherty L.C., Gordon S.M., Seal R.L., Wright M.W., Bruford E.A. (2013). Genenames.org: the HGNC resources in 2013. Nucleic Acids Res..

[5] Ramani A.K., Bunescu R.C., Mooney R.J., Marcotte E.M. (2005). Consolidating the set of known human protein-protein interactions in preparation for large-scale mapping of the human interactome. Genome Biol..

[6] Hart G.T., Ramani A.K., Marcotte E.M. (2006). How complete are current yeast and human protein-interaction networks?. Genome Biol..

[7] Stumpf M.P., Thorne T., de Silva E., Stewart R., An H.J., Lappe M., Wiuf C. (2008). Estimating the size of the human interactome. Proc. Natl. Acad. Sci. USA.

[8] Venkatesan K., Rual J.F., Vazquez A., Stelzl U., Lemmens I., Hirozane-Kishikawa T., Hao T., Zenkner M., Xin X., Goh K.I. (2009). An empirical framework for binary interactome mapping. Nat. Methods.

[9] Tyagi M., Hashimoto K., Shoemaker B.A., Wuchty S., Panchenko A.R. (2012). Large-scale mapping of human protein interactome using structural complexes. EMBO Rep..

[10] Casado-Vela J., Cebrian A., Gomez del Pulgar M.T., Sanchez-Lopez E., Vilaseca M., Menchen L., Diema C., Selles-Marchart S., Martinez-Esteso M.J., Yubero N. (2011). Lights and shadows of proteomic technologies for the study of protein species including isoforms, splicing variants and protein post-translational modifications. Proteomics.

[11] Braun P., Tasan M., Dreze M., Barrios-Rodiles M., Lemmens I., Yu H., Sahalie J.M., Murray R.R., Roncari L., de Smet A.S. (2009). An experimentally derived confidence score for binary protein-protein interactions. Nat. Methods.

[12] Casado-Vela J., Gonzalez-Gonzalez M., Matarraz S., Martínez-Esteso M.J., Vilella M., Sayagues J.M., Fuentes M., Lacal J.C. (2013). Protein arrays: recent achievements and their application to study the human proteome. Curr. Proteomics.

[13] Mishra S. (2012). Computational prediction of protein-protein complexes. BMC Res. Notes.

[14] Jessulat M., Pitre S., Gui Y., Hooshyar M., Omidi K., Samanfar B., Tan le H., Alamgir M., Green J., Dehne F. (2011). Recent advances in protein-protein interaction prediction: Experimental and computational methods. Expert Opin. Drug Discov..

[15] Xia J.F., Wang S.L., Lei Y.K. (2010). Computational methods for the prediction of protein-protein interactions. Protein Pept. Lett..

[16] Skrabanek L., Saini H.K., Bader G.D., Enright A.J. (2008). Computational prediction of protein-protein interactions. Mol. Biotechnol..

[17] Pitre S., Alamgir M., Green J.R., Dumontier M., Dehne F., Golshani A. (2008). Computational methods for predicting protein-protein interactions. Adv. Biochem. Eng. Biotechnol..

[18] Keskin O., Tuncbag N., Gursoy A. (2008). Characterization and prediction of protein interfaces to infer protein-protein interaction networks. Curr. Pharm. Biotechnol..

[19] Gomez S.M., Choi K., Wu Y. (2008). Prediction of protein-protein interaction networks. Curr. Protoc. Bioinformatics.

[20] Fernandez-Suarez X.M., Galperin M.Y. (2013). The 2013 Nucleic Acids Research Database Issue and the online molecular biology database collection. Nucleic Acids Res..

[21] Elefsinioti A., Sarac O.S., Hegele A., Plake C., Hubner N.C., Poser I., Sarov M., Hyman A., Mann M., Schroeder M. (2011). Large-scale de novo prediction of physical protein-protein association. Mol. Cell. Proteomics.

[22] Klingstrom T., Plewczynski D. (2011). Protein-protein interaction and pathway databases, a graphical review. Brief. Bioinform..

[23] Aranda B., Blankenburg H., Kerrien S., Brinkman F.S., Ceol A., Chautard E., Dana J.M., De Las Rivas J., Dumousseau M., Galeota E. (2011). PSICQUIC and PSISCORE: Accessing and scoring molecular interactions. Nat. Methods.

[24] Blankenburg H., Finn R.D., Prlic A., Jenkinson A.M., Ramirez F., Emig D., Schelhorn S.E., Buch J., Lengauer T., Albrecht M. (2009). DASMI: Exchanging, annotating and assessing molecular interaction data. Bioinformatics.

[25] Garcia-Garcia J., Schleker S., Klein-Seetharaman J., Oliva B. (2012). BIPS: BIANA Interolog Prediction Server. A tool for protein-protein interaction inference. Nucleic Acids Res..

[26] Schleker S., Sun J., Raghavan B., Srnec M., Muller N., Koepfinger M., Murthy L., Zhao Z., Klein-Seetharaman J. (2012). The current Salmonella-host interactome. Proteomics Clin. Appl..

[27] Orchard S. (2012). Molecular interaction databases. Proteomics.

[28] National Center for Biotechnology Information. www.ncbi.nlm.nih.gov/gene.

[29] Alzheimer and neuronal disease laboratory_data repository. www.cnb.csic.es/~naranjo/.

[30] Uniprot protein knowledgebase/Swiss-Prot. http://www.uniprot.org/uniprot/.

[31] UniProt Frequently Asked Questions_question_48. http://www.uniprot.org/faq/48.

[32] Uniprot link to CHKA_HUMAN, P35790, Choline kinase. http://www.uniprot.org/uniprot/P35790.

[33] Dubois T., Howell S., Zemlickova E., Aitken A. (2002). Identification of casein kinase Ialpha interacting protein partners. FEBS Lett..

[34] Szklarczyk D., Franceschini A., Kuhn M., Simonovic M., Roth A., Minguez P., Doerks T., Stark M., Muller J., Bork P. (2011). The STRING database in 2011: Functional interaction networks of proteins, globally integrated and scored. Nucleic Acids Res..

[35] String 9.05_web interface. http://string-db.org/.

[36] Psicquic View_web interface. http://www.ebi.ac.uk/Tools/webservices/psicquic/view/main.xhtml.

[37] Casado-Vela J., Cebrian A., Gomez del Pulgar M.T., Lacal J.C. (2011). Approaches for the study of cancer: Towards the integration of genomics, proteomics and metabolomics. Clin. Transl. Oncol..

[38] UniProt Frequently Asked Questions_question_6. www.uniprot.org/faq/6.

[39] Gene Symbol Redundancy Checker. https://dl.dropboxusercontent.com/u/77276631/SymbolRedundancy.zip.

[40] Swiss-Prot incorporated the International Protein Index Database. http://www.uniprot.org/news/2011/05/03/release.

[41] Navarro-Munoz M., Ibernon M., Bonet J., Perez V., Pastor M.C., Bayes B., Casado-Vela J., Navarro M., Ara J., Espinal A. (2012). Uromodulin and alpha(1)-antitrypsin urinary peptide analysis to differentiate glomerular kidney diseases. Kidney Blood Press. Res..

[42] Tamm I., Horsfall F.L. (1952). A mucoprotein derived from human urine which reacts with influenza, mumps, and Newcastle disease viruses. J. Exp. Med..

[43] Klein J., Jupp S., Moulos P., Fernandez M., Buffin-Meyer B., Casemayou A., Chaaya R., Charonis A., Bascands J.L., Stevens R. (2012). The KUPKB: A novel Web application to access multiomics data on kidney disease. FASEB J..

[44] Casado-Vela J., del Pulgar T.G., Cebrian A., Alvarez-Ayerza N., Lacal J.C. (2011). Human urine proteomics: Building a list of human urine cancer biomarkers. Expert Rev. Proteomics.

[45] Hegde S.R., Manimaran P., Mande S.C. (2008). Dynamic changes in protein functional linkage networks revealed by integration with gene expression data. PLoS Comput. Biol..

[46] Buxbaum J.D., Choi E.K., Luo Y., Lilliehook C., Crowley A.C., Merriam D.E., Wasco W. (1998). Calsenilin: A calcium-binding protein that interacts with the presenilins and regulates the levels of a presenilin fragment. Nat. Med..

[47] Carrion A.M., Link W.A., Ledo F., Mellstrom B., Naranjo J.R. (1999). DREAM is a Ca^2+^-regulated transcriptional repressor. Nature.

[48] An W.F., Bowlby M.R., Betty M., Cao J., Ling H.P., Mendoza G., Hinson J.W., Mattsson K.I., Strassle B.W., Trimmer J.S., Rhodes K.J. (2000). Modulation of A-type potassium channels by a family of calcium sensors. Nature.

[49] Mellstrom B., Naranjo J.R. (2001). Ca^2+^-dependent transcriptional repression and derepression: DREAM, a direct effector. Semin. Cell Dev. Biol..

[50] Pathguide.org. http://www.pathguide.org/.

[51] Rivas M., Villar D., Gonzalez P., Dopazo X.M., Mellstrom B., Naranjo J.R. (2011). Building the DREAM interactome. Sci. China Life Sci..

[52] Fedrizzi L., Lim D., Carafoli E., Brini M. (2008). Interplay of the Ca^2+^-binding protein DREAM with presenilin in neuronal Ca^2+^ signaling. J. Biol. Chem..

[53] Ledo F., Carrion A.M., Link W.A., Mellstrom B., Naranjo J.R. (2000). DREAM-alphaCREM interaction via leucine-charged domains derepresses downstream regulatory element-dependent transcription. Mol. Cell. Biol..

[54] Rivas M., Aurrekoetxea K., Mellstrom B., Naranjo J.R. (2011). Redox signaling regulates transcriptional activity of the Ca^2+^-dependent repressor DREAM. Antioxid. Redox Signal..

[55] Ledo F., Kremer L., Mellstrom B., Naranjo J.R. (2002). Ca^2+^-dependent block of CREB-CBP transcription by repressor DREAM. EMBO J..

[56] Rivas M., Mellstrom B., Naranjo J.R., Santisteban P. (2004). Transcriptional repressor DREAM interacts with thyroid transcription factor-1 and regulates thyroglobulin gene expression. J. Biol. Chem..

[57] Scsucova S., Palacios D., Savignac M., Mellstrom B., Naranjo J.R., Aranda A. (2005). The repressor DREAM acts as a transcriptional activator on Vitamin D and retinoic acid response elements. Nucleic Acids Res..

[58] Lusin J.D., Vanarotti M., Li C., Valiveti A., Ames J.B. (2008). NMR structure of DREAM: Implications for Ca(2+)-dependent DNA binding and protein dimerization. Biochemistry.

[59] Zaidi N.F., Kuplast K.G., Washicosky K.J., Kajiwara Y., Buxbaum J.D., Wasco W. (2006). Calsenilin interacts with transcriptional co-repressor C-terminal binding protein(s). J. Neurochem..

[60] Ruiz-Gomez A., Mellstrom B., Tornero D., Morato E., Savignac M., Holguin H., Aurrekoetxea K., Gonzalez P., Gonzalez-Garcia C., Cena V. (2007). G protein-coupled receptor kinase 2-mediated phosphorylation of downstream regulatory element antagonist modulator regulates membrane trafficking of Kv4.2 potassium channel. J. Biol. Chem..

[61] Quintero C.A., Valdez-Taubas J., Ferrari M.L., Haedo S.D., Maccioni H.J. (2008). Calsenilin and CALP interact with the cytoplasmic tail of UDP-Gal:GA2/GM2/GD2 beta-1,3-galactosyltransferase. Biochem. J..

[62] Rivas M., Mellstrom B., Torres B., Cali G., Ferrara A.M., Terracciano D., Zannini M., Morreale de Escobar G., Naranjo J.R. (2009). The DREAM protein is associated with thyroid enlargement and nodular development. Mol. Endocrinol..

[63] Wu L.J., Mellstrom B., Wang H., Ren M., Domingo S., Kim S.S., Li X.Y., Chen T., Naranjo J.R., Zhuo M. (2010). DREAM (downstream regulatory element antagonist modulator) contributes to synaptic depression and contextual fear memory. Mol. Brain.

[64] Anderson D., Mehaffey W.H., Iftinca M., Rehak R., Engbers J.D., Hameed S., Zamponi G.W., Turner R.W. (2010). Regulation of neuronal activity by Cav3-Kv4 channel signaling complexes. Nat. Neurosci..

[65] Zhang Y., Su P., Liang P., Liu T., Liu X., Liu X.Y., Zhang B., Han T., Zhu Y.B., Yin D.M. (2010). The DREAM protein negatively regulates the NMDA receptor through interaction with the NR1 subunit. J. Neurosci..

[66] Palczewska M., Casafont I., Ghimire K., Rojas A.M., Valencia A., Lafarga M., Mellstrom B., Naranjo J.R. (2010). Sumoylation regulates nuclear localization of repressor DREAM. Biochim. Biophys. Acta.

[67] Ramachandran P.L., Craig T.A., Atanasova E.A., Cui G., Owen B.A., Bergen H.R., Mer G., Kumar R. (2012). The potassium channel interacting protein 3 (DREAM/KChIP3) heterodimerizes with and regulates calmodulin function. J. Biol. Chem..

[68] Full list of human genes, acronyms and descriptions (NCBI). ftp://ftp.ncbi.nih.gov/gene/DATA/GENE_INFO/Mammalia/.

[69] DASMI_web interface. http://dasmi.de/dasmiweb.php.

[70] BIPS_web interface. http://sbi.imim.es/web/index.php/research/servers/bips..

